# Application of the Asthma Phenotype Algorithm from the Severe Asthma Research Program to an Urban Population

**DOI:** 10.1371/journal.pone.0044540

**Published:** 2012-09-13

**Authors:** Paru Patrawalla, Angeliki Kazeros, Linda Rogers, Yongzhao Shao, Mengling Liu, Maria-Elena Fernandez-Beros, Shulian Shang, Joan Reibman

**Affiliations:** 1 Division of Pulmonary and Critical Care Medicine, Department of Medicine, New York University School of Medicine, New York, New York, United States of America; 2 Environmental Medicine, New York University School of Medicine, New York, New York, United States of America; Leiden University Medical Center, The Netherlands

## Abstract

**Rationale:**

Identification and characterization of asthma phenotypes are challenging due to disease complexity and heterogeneity. The Severe Asthma Research Program (SARP) used unsupervised cluster analysis to define 5 phenotypically distinct asthma clusters that they replicated using 3 variables in a simplified algorithm. We evaluated whether this simplified SARP algorithm could be used in a separate and diverse urban asthma population to recreate these 5 phenotypic clusters.

**Methods:**

The SARP simplified algorithm was applied to adults with asthma recruited to the New York University/Bellevue Asthma Registry (NYUBAR) to classify patients into five groups. The clinical phenotypes were summarized and compared.

**Results:**

Asthma subjects in NYUBAR (n = 471) were predominantly women (70%) and Hispanic (57%), which were demographically different from the SARP population. The clinical phenotypes of the five groups generated by the simplified SARP algorithm were distinct across groups and distributed similarly to those described for the SARP population. Groups 1 and 2 (6 and 63%, respectively) had predominantly childhood onset atopic asthma. Groups 4 and 5 (20%) were older, with the longest duration of asthma, increased symptoms and exacerbations. Group 4 subjects were the most atopic and had the highest peripheral eosinophils. Group 3 (10%) had the least atopy, but included older obese women with adult-onset asthma, and increased exacerbations.

**Conclusions:**

Application of the simplified SARP algorithm to the NYUBAR yielded groups that were phenotypically distinct and useful to characterize disease heterogeneity. Differences across NYUBAR groups support phenotypic variation and support the use of the simplified SARP algorithm for classification of asthma phenotypes in future prospective studies to investigate treatment and outcome differences between these distinct groups.

**Trial Registration:**

Clinicaltrials.gov NCT00212537

## Introduction

Asthma affects more than 17 million American adults, and is estimated to cost $20.7 billion [1], [2]. Despite guideline-recommended treatment strategies, asthma morbidity remains high, with almost 50% of adults reporting an exacerbation in the previous year [3]. Asthma is characterized by chronic inflammation, variable symptoms and airflow limitation [4]. However, asthma is heterogeneous and appropriate classification of asthma phenotypes using clinical characteristics and immunologic biomarkers can improve our understanding of asthma pathogenesis, therapeutics and targeted management.

Cluster analysis, incorporating demographic, clinical and biologic variables, has recently been used to identify distinct asthma phenotype groups [5], [6]. The Severe Asthma Research Program (SARP) performed unsupervised cluster analysis of subjects with mild to severe persistent asthma using 34 variables and identified five clusters. These clusters had differences in gender, asthma onset, lung function, atopic status, asthma control and healthcare utilization (HCU) [6]. The different characteristics of the clusters suggests potential differences in pathophysiology between distinct clusters. However, application of cluster analysis to clinical settings or to prospective studies on asthma is limited by the complexity of cluster analysis and multiplicity of variables. Further analysis of the SARP population led to a simple classification rule using only three of 34 variables which could successfully assign their subjects into the five defined clusters with about 80% accuracy [6]. These variables included: baseline percent of predicted forced expiratory volume in one second (%predicted FEV_1_), maximal %predicted FEV_1_, and age of asthma onset. This simplified SARP algorithm for classification uses information on three variables that is readily available in the clinical setting, however the clinical utility of this simplified algorithm and the stability of the resulting five groups have not been examined in a separate and diverse asthma population.

The NYUBAR is a registry of adults with asthma recruited from an urban population in New York City [7], [8]. The registry includes a predominance of women and has a diverse distribution of race/ethnicity. The diversity of the NYUBAR population makes it ideal to test the robustness of the simplified SARP algorithm. We hypothesized that application of the simplified SARP algorithm to the NYUBAR asthma registry would produce five groups where most of the significant elements of the distinct phenotypes identified in SARP are preserved [6]. We report that application of the simplified SARP algorithm to an urban population reproduced groups with similar phenotypic characteristics to those reported for the SARP population. In addition, differences in biomarkers across the five groups are reported. Separate cluster analysis of the NYUBAR resulted in five clusters that were phenotypically similar, although not identical to those in SARP.

## Methods

### Study design and subject recruitment

Subjects with asthma were identified from the NYUBAR in New York City. All participants signed informed consent to enroll in this registry (approved by the New York University School of Medicine Institutional Review Board, H-9698). Asthma cases were referred to the registry by the Bellevue Hospital Center Asthma Clinic and local clinics. Subjects were excluded if they were <18 or >75 years old, former >10 pack-year or current smokers, had unstable cardiac disease, uncontrolled hypertension, lung disease other than asthma, or neuromuscular disease. Details of subject recruitment have been reported previously [7], [8].

Questionnaires, lung function testing, eosinophil counts, allergen-specific and total IgE levels were all completed on the same day. All subjects were seen by a clinician with experience in asthma diagnosis and management. Subjects were determined to have a diagnosis of “asthma” based on a standardized questionnaire and definition modified from international studies of asthma [9], [10]. Since most of these adult patients were using chronic medication for asthma or had longstanding disease, a 12% change in FEV_1_ or bronchial hyperresponsiveness with methacholine challenge testing was not used as a criterion for diagnosis. The diagnosis of asthma was further confirmed using the published algorithm of Enright et al [11].

### Questionnaires

Socio-demographic factors, asthma control and severity, medication use and health care utilization (HCU) were based on self-report from a standardized questionnaire. Functional status was assessed by the number of city blocks a subject could walk before stopping with shortness of breath, a quantifiable urban activity. Asthma exacerbations and HCU were examined as outcomes and quantified as the number of oral corticosteroid courses (OCS), emergency department (ED) visits and hospitalizations (HA) reported by each subject in the previous year [12].

### Allergy Testing

Measurements of total serum IgE and specific IgE for allergens significant for the Northeastern United States were performed in a commercial laboratory (Pharmacia ImmunoCAP assay; Quest Diagnostics; Teterboro, NJ). Allergen-specific IgE level ≥0.35 kilo-international units (kIU)/L was considered positive. Atopy was defined as at least one elevated allergen-specific IgE. Indoor allergens included cat and dog dander, *blatella germanica* (cockroach) and *dermatophagoides pteronyssinus* (house dust mite). Outdoor allergens included maple, ragweed, birch, elm, oak and ash and molds included *aspergillus fumigatus* and *alternaria alternata*.

### Spirometry

Pre- and post-bronchodilator (bd) spirometry were performed according to American Thoracic Society guidelines [13] and normal values were obtained from Hankinson et al [14]. Post-bd spirometry was obtained after albuterol sulfate (180 mcg) administration. If the subject was not on maximal asthma therapy with an FEV_1_<80% predicted, treatment was escalated and spirometry repeated, with the best values reported.

### Application of the simplified SARP algorithm for asthma phenotypes

A three-variable algorithm developed in the SARP population was used to classify the NYUBAR asthma patients into five groups [6]. These 3 variables are: baseline %predicted FEV_1_, maximal % predicted FEV_1_, and age of asthma onset [6] We applied the simplified SARP algorithm to our population by creating a formula using these variables to assign NYUBAR subjects to one of five groups. Age of asthma onset was defined by self-report of physician diagnosis or onset of asthma symptoms if physician diagnosis was unknown. Baseline %predicted FEV_1_ was defined as %predicted pre-bronchodilator FEV_1_ (pre-FEV_1_), and maximal FEV_1_ was defined as %predicted post-bronchodilator FEV_1_ (post-FEV_1_) after the administration of albuterol sulfate (180 mcg). As defined by the simplified SARP algorithm, the five groups were classified as: Group 1, pre-FEV_1_≥68%, post-FEV_1_≥108%; Group 2, pre-FEV_1_≥68%, post-FEV_1_<108% and asthma onset <40 years; Group 3, pre-FEV_1_≥68%, post-FEV_1_<108% and asthma onset ≥40 years; Group 4, pre-FEV_1_<68%, post-FEV_1_≥65%; Group 5, pre-FEV_1_<68%, post-FEV_1_<65%.

### Statistical Analysis

To compare differences between groups, summary statistics are reported as median values and ranges for continuous variables, and as percentages and counts for categorical variables. Geometric means and ranges are reported for total IgE and percentage peripheral blood eosinophils. Univariate analysis of associations was performed using the χ^2^ test for categorical variables, ANOVA test for normally distributed continuous variables, and the non-parametric Kruskal-Wallis tests for other continuous variables. *P* values≤0.05 were considered statistically significant. For the cluster analysis, Ward's minimum-variance hierarchical clustering method with standardization of incorporated variables was performed using R 2.12.0. Analyzed variables include demographics, lung function related variables, atopy, medication use, etc. A complete list of variables is given in the Appendix S1.

## Results

### Subject characteristics

Between April 2002 and October 2009, 496 asthma subjects underwent standardized evaluations as part of the NYUBAR. Of these, 25 individuals were excluded: 23 had incomplete spirometry data and/or age of asthma onset and two had markedly elevated IgE or blood eosinophils suggesting an alternative diagnosis. After exclusions, 471 asthma subjects were available for analysis and their characteristics are shown in Table 1. Median age was 39 years, 70% of subjects were female and 80% reported an annual income below $50,000. The population had a diverse race/ethnicity with 57% of subjects identifying themselves as Hispanic, the majority reporting Puerto Rican or Dominican ethnicity. More than one-third of all subjects were obese, and nearly 20% were former <10 pack-year smokers.

**Table 1:** Subject characteristics of NYUBAR asthma cases (N = 471).

Characteristics:	
Age: median (IQR)	39 (27–51)
Gender, N (%)	
Female	328 (70)
Male	143 (30)
Self-reported Race/Ethnicity, N (%)	
Hispanic†	267 (57)
Non-Hispanic Black	72 (15)
Non-Hispanic White	95 (20)
Asian/Other	37 (8)
BMI>30, N (%)	171 (36)
Income <$50,000, N (%)*	319 (80)
Former smoker <10 p-y, N (%)*	91 (19)

* Data on income missing on 73 subjects, we report percentage for only those reported; data on former smoking status missing on 1 subject.

† Of 267 subjects self-reporting ethnicity as Hispanic, 97% (n = 260) of subjects reported White race and 3% (n = 7) reported Black race.

### Subject characteristics across NYUBAR groups after application of the simplified SARP algorithm

The simplified SARP algorithm was applied to create 5 groups (Table 2). Analysis of these groups revealed that few subjects fell into Group 1 (n = 26) and most were in Group 2 (n = 299). Groups 3, 4 and 5 were of relatively equal size (n = 46, 51, 49 respectively). Groups differed by age at enrollment into the NYUBAR, and Groups 3 and 5 were the oldest (median 54 and 50 years respectively). Women were prevalent in all groups; however Groups 1 and 5 had the least women. Differences in race/ethnicity were also noted across groups. Group 3 had the highest rate of obesity. There were no differences in annual income or former smoking status. Group 3 had the highest percentage of subjects reporting gastroesophageal reflux symptoms (GERD) or hypertension (HTN). Although Groups 3 and 5 had the oldest subjects, Groups 4 and 5 had the longest duration of asthma (median 24 and 32 years respectively). Group 5 had the highest rate (16%) of previous intubations.

**Table 2:** Subject characteristics in NYUBAR groups (N = 471).

	Group 1	Group 2	Group 3	Group 4	Group 5	p value
	N = 26	N = 299	N = 46	N = 51	N = 49	
Age at enrollment, median (IQR)	29 (23–44)	33 (24–45)	54 (48–59)	44 (35–55)	50 (41–62)	<0.0001
Gender, N (%)						0.02
Female	15 (58)	221 (74)	34 (74)	32 (63)	26 (53)	
Male	11 (42)	78 (26)	12 (26)	19 (37)	23 (47)	
Self-reported Race/Ethnicity, N (%)						0.03
Hispanic†	10 (38)	174 (58)	26 (57)	33 (65)	24 (49)	
Non-Hispanic Black	6 (23)	46 (15)	4 (9)	8 (16)	8 (16)	
Non-Hispanic White	9 (35)	60 (20)	14 (30)	4 (8)	8 (16)	
Asian	1 (4)	18 (6)	1 (2)	5 (10)	9 (18)	
Other	0 (0)	1 (.3)	1 (2)	1 (2)	0 (0)	
BMI>30, N (%	3 (12)	102 (34)	22 (48)	23 (45)	21 (43)	0.0007
Former smoker <10 p-y, N (%)	4 (15)	62 (21)	9 (20)	8 (16)	8 (16)	NS
Reported co-morbidity, N (%)*						
Gastroesophageal reflux	2 (8)	76 (26)	21 (47)	10 (20)	15 (31)	0.003
Hypertension	1 (4)	41 (14)	21 (47)	13 (27)	20 (41)	<0.0001
Diabetes	1 (4)	17 (6)	7 (15)	7 (14)	9 (18)	0.01
Eczema	3 (12)	45 (15)	2 (4)	11 (22)	7 (16)	NS
Age of asthma onset, median (IQR)	12 (6–25)	12 (5–24)	46 (43–53)	18 (6–31)	20 (6–30)	<0.0001
Years with asthma, median (IQR)	17 (9–21)	18 (10–31)	5 (1–11)	24 (12–34)	32 (17–42)	<0.0001
Ever intubated (lifetime), N (%)*	0 (0)	31 (11)	1 (2)	6 (12)	8 (16)	0.02

* Data missing for GERD in 8 subjects, hypertension in 9 subjects, diabetes in 5 subjects, eczema in 8 subjects; data missing for ever intubated in 8 subjects.

† Of 267 subjects self-reporting ethnicity as Hispanic, 3% (n = 7) of subjects reported Black race: 5 subjects were in Group 2 and 2 subjects were in Group 5.

### Spirometry and asthma onset across NYUBAR groups

After application of the formula using the three variables, pre- and post-FEV_1_ were highest in Group 1 and values decreased across the groups (Table 3). Age of asthma onset was earliest in groups 1 and 2, with late childhood or early adult onset in Groups 4 and 5, and adult onset in Group 3 (Table 2). These distributions were expected based on the variables that were used, and confirmed the appropriate development of the formulas for the simplified SARP algorithm. Additional analysis of lung function revealed that FEV_1_/FVC and % predicted FVC were also highest in Group 1 and decreased across groups. Furthermore, although most participants used a controller medication, a bronchodilator response was noted in some, with Group 4 having the greatest response.

**Table 3:** Lung function in NYUBAR groups (N = 471).

Lung function, mean (SE)	Group 1	Group 2	Group 3	Group 4	Group 5	p value
	N = 26	N = 299	N = 46	N = 51	N = 49	
% predicted pre-FEV_1_	108 (1.8)	84 (0.5)	82 (1.4)	62 (1.3)	47 (1.3)	<0.0001
% predicted post-FEV_1_	113 (1.8)	89 (0.5)	85 (1.3)	72 (1.3)	53 (1.3)	<0.0001
FEV_1_/FVC	81 (1.5)	78 (0.4)	77 (1.1)	68 (1)	62 (1.1)	<0.0001
% predicted FVC	111 (2.0)	90 (0.6)	84 (1.5)	74 (1.4)	59 (1.5)	<0.0001
Change in FEV_1_, mL	180 (33)	137 (10)	86 (25)	309 (23)	187 (24)	<0.0001
Change in FEV_1_, %	5.5 (1.0)	4.2 (0.3)	2.8 (0.7)	9.9 (0.7)	6.3 (0.7)	<0.0001

### Asthma exacerbations and control across NYUBAR groups

**Figure 1:**
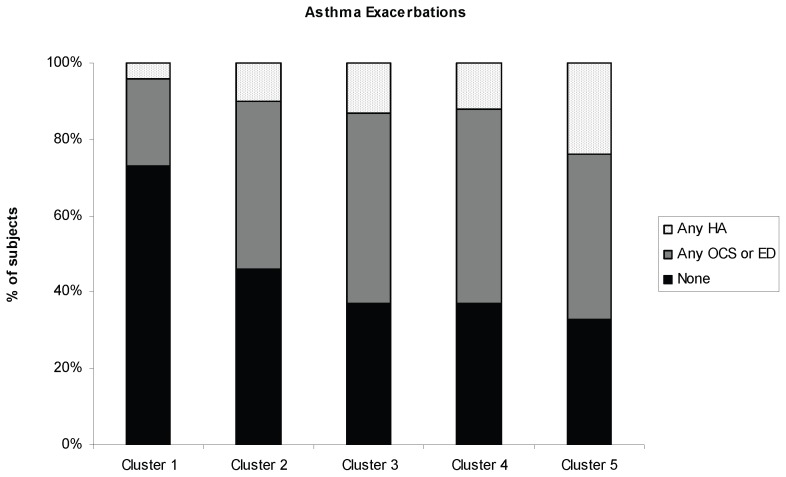
Asthma exacerbations and healthcare utilization (HCU) for NYUBAR groups in the year prior to study enrollment. No exacerbation is shown in black; any OCS or ED visit is shown in dark gray; and any HA is shown in light gray. Data missing in 11 subjects; (p value = .02, χ^2^ test).

Asthma control was assessed using frequency of daytime and nocturnal asthma symptoms, use of short acting bronchodilator (SABA) and asthma medication use. Functional status was quantified as the number of city blocks a subject could walk before needing to stop for shortness of breath. Symptoms, medication use and functional status differed across the 5 groups. Although Groups 2, 3, 4 and 5 all had a high percentage of individuals reporting uncontrolled daytime symptoms, Group 4 had the highest percentage of subjects with uncontrolled daytime and nocturnal symptoms and the worst functional status, with 64% limited to walking <10 city blocks (equivalent of approximately one half mile) before stopping with shortness of breath. Twenty percent of Group 1 subjects used an inhaled corticosteroid (ICS), whereas 90% of Group 5 subjects used an ICS, with a majority on at least 2 controller medications. Few subjects in Groups 2, 4 and 5, and none in Groups 1 and 3 used chronic daily oral corticosteroids (Table 4). Asthma exacerbations and HCU differed across the groups (Figure 1). Nearly 75% of Group 1 subjects reported no asthma exacerbation as defined by any OCS, ED visit or HA in the year prior. In contrast, >50% of Group 2 reported an asthma exacerbation. Groups 3, 4 and 5 had high rates of exacerbations with only 37% of subjects in Groups 3 and 4, and 33% of subjects in Group 5 reporting no exacerbations in the previous year. Group 5 had the highest percentage of exacerbations, including at least 1 HA in 24% in the previous year.

**Table 4:** Asthma control in NYUBAR groups (N = 471).

Asthma control, N (%)	Group 1	Group 2	Group 3	Group 4	Group 5	p-value
	N = 26	N = 299	N = 46	N = 51	N = 49	
Daytime symptoms≥2 days/week	11 (42)	189 (63)	35 (76)	40 (78)	32 (65)	0.01
Nocturnal symptoms≥2 days/week	5 (19)	98 (33)	19 (41)	28 (55)	15 (31)	0.01
SABA use ≥2 days/week	4 (15)	82 (28)	14 (30)	19 (40)	19 (43)	NS
Walk <10 city blocks	3 (12)	93 (32)	20 (43)	32 (64)	23 (49)	<0.0001
Medication use†						<0.0001
No controller	20 (77)	121 (40)	11 (24)	11 (22)	5 (10)	
Non-ICS controller alone*	1 (4)	9 (3)	3 (7)	2 (4)	0 (0)	
ICS alone	3 (12)	51 (17)	10 (22)	3 (6)	3 (6)	
ICS+LABA	2 (8)	69 (23)	14 (30)	18 (35)	24 (49)	
ICS+other**	0 (0)	8 (3)	1 (2)	2 (4)	3 (6)	
ICS+LABA+other**	0 (0)	39 (13)	7 (15)	14 (27)	11 (22)	
Oral corticosteroid use	0 (0)	2 (1)	0 (0)	1(2)	3(6)	

Data missing for daytime symptoms on 1 subject, nocturnal symptoms in 2 subjects, SABA use in 15 subjects, functional status on 12 subjects.

† Only 2 subjects used omalizumab: 1 in Group 2 and 1 in Group 3.

* Non-ICS controller includes theophylline, leukotriene inhibitors, cromolyn and LABA.

** other controller includes theophylline, leukotriene inhibitors, and cromolyn.

### Peripheral biomarkers across NYUBAR groups

Peripheral eosinophils, total and allergen-specific IgE differed across groups (Table 5). Groups 1 and 3 had the lowest percentages of eosinophils and total IgE was also lowest in Group 3. Atopy was common in all groups; however fewer individuals in Group 3 had an allergen-specific IgE. Group 4 had the highest percentage peripheral eosinophils, total IgE and number of atopic subjects including subjects with any indoor allergen-specific IgE. Group 1 had the highest percentage of subjects with any outdoor allergen-specific IgE.

**Table 5:** Biomarkers in NYUBAR groups (N = 471).

	Group 1	Group 2	Group 3	Group 4	Group 5	p value
	N = 26	N = 299	N = 46	N = 51	N = 49	
% Peripheral eosinophils, geometric mean (SE)	2.5 (1.2)	3.2 (1.1)	2.5 (1.1)	3.98 (1.1)	3.2 (1.1)	0.004
Total IgE (IU/mL), geometric mean (SE)	100 (1.3)	126 (1.1)	50 (1.3)	200 (1.3)	100 (1.3)	0.0005
Any allergen-specific IgE, N (%)	21 (81)	234 (79)	26 (57)	44 (86)	35 (71)	0.009
Any indoor allergen-specific IgE, N (%)	19 (73)	215 (72)	18 (40)	42 (82)	33 (67)	0.0002
Any outdoor allergen-specific IgE, N (%)	17 (65)	136 (46)	14 (31)	25 (49)	18 (37)	0.045
Any mold allergen-specific IgE, N (%)	5 (19)	54 (18)	4 (9)	7 (14)	12 (24)	NS

Data missing on % peripheral eosinophils in 33 subjects, total IgE in 1 subject and some or all data on allergen-specific IgE in 2 subjects.

### Cluster analysis

Our goal was to assess whether we could apply a previously developed algorithm to our population and obtain results that were similar to those reported for the SARP population. We also evaluated whether a separate cluster analysis of the diverse NYUBAR urban population would result in clusters that were similar to those in SARP. Twenty seven variables on demographics, lung function, medication use, etc. were used for cluster analysis (see Appendix S1). Only 27 variables are used because some of the variables used in the SARP analysis were not measured in NYUBAR. Since the NYUBAR population was predominantly female, we omitted gender as a variable. Unsupervised hierarchical clustering with the defined variables resulted in 5 clusters of similar, although not identical proportions. Given that the NYUBAR asthma patients are quite different from the SARP asthma patients in demographics, and the cluster variables used are not identical for the two patient cohorts, this is not an unexpected finding. Nevertheless, NYUBAR Clusters 2, 3 and 5 had the greatest number of individuals. The clusters differed significantly from each other by clinical characteristics with NYUBAR Clusters 3 and 5 containing the most female and oldest participants; race/ethnicity differed among the clusters, with the lowest proportion of Hispanic participants in Cluster 1. NYUBAR Clusters 1, 2 and 4 had early onset of asthma, whereas 3 and 5 had adult onset. NYUBAR Clusters 4 and 5 had the longest duration of asthma. As seen after clustering of the SARP population, lung function, as measured by pre- and post-FEV_1_, declined across NYUBAR clusters with the best values in NYUBAR Cluster 1 and the most severe obstruction in Clusters 4 and 5. However, the differences in lung function were not as extreme as those defined for the simplified SARP algorithm. Similar to the SARP distribution, NYUBAR Clusters 4 and 5 had the worst asthma control and most HCU. Distribution of total IgE was also similar to that described in SARP, with elevated levels of total IgE and presence of atopy most common in Clusters 1 and 4 and lowest in Cluster 3.

## Discussion

Application of the simplified SARP algorithm to the separate and demographically diverse NYUBAR population revealed 5 groups phenotypically similar to those identified in SARP. This algorithm has not previously been tested in populations that differed from SARP, thus our findings support the use of the algorithm for separate asthma populations. In addition, biomarkers were not used in the cluster analysis performed by SARP; however differences in the distribution of peripheral eosinophils as well as total and allergen-specific IgE were detected across NYUBAR groups, supporting phenotypic variation. A separate cluster analysis of the NYUBAR population resulted in clusters that were qualitatively similar to those described for SARP, supporting the robustness of the clinical phenotypes defined by the SARP cluster analysis and the simplified SARP algorithm for classification.

Current guidelines rely on severity classification defined by symptoms and pulmonary function to separate asthma patients into similarly managed groups [4], [15]. This classification scheme ignores asthma subphenotypes that cross severity levels and may have different treatment responses, thus characterizing them can have clinical implications [16]. Using a complex unsupervised cluster analysis, 5 subphenotypes of asthma were identified in the SARP population [6]. A simplified algorithm was suggested to reproduce the 5 subphenotypes. The NYUBAR is a diverse urban population that is predominately female and largely Hispanic. As such, it differs from the SARP population. Despite population differences, our study supported the finding that the simplified SARP algorithm can distinguish these 5 groups in a separate population.

There were many similarities between the NYUBAR groups and SARP clusters. NYUBAR Group 1 and 2 subjects had early onset atopic asthma and normal lung function. The NYUBAR group 1 was young with little comorbidity and had the best functional status and asthma control. The NYUBAR group 2 was the largest group (63%) and was predominately female and Hispanic.

NYUBAR group 3 was a unique group of older, obese, and predominately female subjects. They had adult-onset asthma with frequent comorbidities such as GERD and HTN. Despite only moderate reduction in lung function without bronchodilator response, many subjects reported uncontrolled daytime symptoms, reduced functional status, and moderate HCU. Peripheral eosinophils were not elevated in this group, and fewer subjects were atopic. This group was similar to that described in SARP and with the group of obese subjects with late-onset asthma who were recently shown to have less airway obstruction, bronchodilator responsiveness, and atopy when compared to obese subjects with early onset of asthma [6], [17].

NYUBAR groups 4 and 5 included older subjects with late adolescent-onset and long duration of asthma. They were obese with frequent comorbidities. Despite frequent controller use, both Groups 4 and 5 reported frequent daytime symptoms, poor functional status and had severe reductions in lung function. NYUBAR group 4 retained a bronchodilator response resulting in near normal lung function. Groups 4 and 5 had the highest HCU, with over 60% reporting an exacerbation in the previous year requiring OCS, ED visit or HA. These findings are similar to those in SARP [6]. In contrast to SARP, NYUBAR group 4 subjects were a predominately female Hispanic population with late adolescent-onset of asthma. Group 5 represented a unique population of older subjects with long asthma duration, and reduced lung function despite bronchodilator or controller use, consistent with persistent obstruction. Long duration of asthma has previously been shown to be associated with increased airflow limitation and hyperinflation in elderly asthmatics [6], [18].

Peripheral eosinophils, total and allergen-specific IgE differed significantly in the NYUBAR groups. This finding supports phenotypic variation across groups as these variables were not used in the cluster analysis performed by SARP [6] Notably, Group 4 subjects had the highest peripheral eosinophils and total IgE as well as a high rate of indoor allergen-specific IgE. Chronic exposure to indoor allergens in sensitized patients is associated with asthma morbidity in some, but not all studies of inner-city adults [19], [20], [21] and Group 4 subjects had poor asthma control and increased exacerbations. Although Groups 1 and 2 also had a high percentage of atopic individuals, many had elevated outdoor-allergen-specific IgE, suggesting that Groups 1, 2 and 4 reflect a spectrum of disease with atopic asthma. NYUBAR group 3 was the least atopic, similar to the SARP population [6]. In addition, few subjects in Group 3 had elevated peripheral eosinophils.

The ability to identify groups using a simplified algorithm has potential clinical significance. Identification of those with milder atopic disease (Groups 1 and 2) might lead to interventions that differ from those with later onset disease and comorbid conditions (Cluster 3). Chronic persistent obstruction in asthma has been associated with ongoing inflammation and an increase in airway smooth muscle [22]. The identification of phenotypic subgroups with potential for these pathologic changes (Groups 4 and 5) might suggest interventions to target inflammation, although the focus on allergic components might differ between the two.

There are several potential limitations to our study. We used a clinical diagnosis of asthma and did not perform methacholine challenge testing in subjects that did not meet bronchodilator criteria for asthma, a difference in definition from SARP that is most prominent in Group 3. Despite this difference, we have shown that the NYUBAR groups are phenotypically similar to SARP, suggesting that the simplified SARP algorithm can be applied to subjects with asthma who are using chronic medication [6]. We did not have a measurement of post-bronchodilator spirometry that was identical to that in SARP. Our post-bronchodilator testing was performed after inhalation of 180 mcg of albuterol sulfate rather than after an amount required to obtain a maximal FEV_1_ as described for SARP. Again, despite this difference, the application of the simplified SARP algorithm using this variable resulted in similar groups. NYUBAR Group 1 was the smallest (6%), differing from SARP. Differences in group size distribution may be due to recruitment bias as NYUBAR focused on a clinical program of patients with uncontrolled asthma. Despite this, the NYUBAR population separated into groups phenotypically similar to those in SARP [6].

Our data suggest that application of the simplified SARP algorithm to a demographically different population can yield groups phenotypically similar to those described for SARP, including a Group 3 that is distinct from other groups. Importantly, we noted differences in the distribution of biomarkers across NYUBAR groups, further supporting important phenotypic differences in these 5 clusters. The data support the use of this simplified algorithm for classification in future prospective studies to investigate treatment and outcome differences between these distinct groups.

## Supporting Information

Appendix S1 **List of variables used in cluster analysis.**
(DOC)
