# Supplementary material for: Application of the Asthma Phenotype Algorithm from the Severe Asthma Research Program to an Urban Population
Source: PLoS One. 2012 Sep 13;7(9):e44540. doi: 10.1371/journal.pone.0044540 (PMC3441500; doi:10.1371/journal.pone.0044540)
Supplement: Appendix S1 — List of variables used in cluster analysis. (DOC) [file pone.0044540.s001.doc]

Appendix S1: List of variables used in cluster analysis

| Variable  Number | Variable Name |
| --- | --- |
| 1 | Race/Ethnicity |
| 2 | BMI |
| 3 | Age at enrollment |
| 4 | Age at asthma onset |
| 5 | Asthma duration |
| 6 | Baseline percent predicted FEV_1_ |
| 7 | Baseline percent predicted FVC |
| 8 | FEV_1_/FVC |
| 9 | Post-bronchodilator percent predicted FEV_1_ |
| 10 | Post-bronchodilator percent predicted FVC |
| 11 | Post-bronchodilator percent change in FEV_1_ |
| 12 | Number of allergen-specific IgE |
| 13 | Inhaled corticosteroid use (composite) |
| 14 | Total number of controllers (composite) |
| 15 | Types of controllers (composite) |
| 16 | Healthcare utilization past year (composite) |
| 17 | History of intubation in lifetime |
| 18 | Symptom score over last two weeks |
| 19 | Cat/dog allergen specific IgE (composite) |
| 20 | Dust mite/cockroach allergen specific IgE (composite) |
| 21 | Mold allergen specific IgE (composite) |
| 22 | Pollen allergen specific IgE (composite) |
| 23 | Allergic rhinitis/hay fever |
| 24 | Nasal polyps |
| 25 | Sinusitis |
| 26 | GERD/HTN (composite) |
| 27 | Remote smoking history (< 10 p-y) |
